# Enzymatic Acylation of Black Rice Anthocyanins and Evaluation of Antioxidant Capacity and Stability of Their Derivatives

**DOI:** 10.3390/foods12244505

**Published:** 2023-12-17

**Authors:** Yue Kong, Xinhui Wang, Zenan Wu, Yanhui Li, Fu Xu, Fengying Xie

**Affiliations:** College of Food Science, Northeast Agricultural University, Heilongjiang150030, China; kyyy0531@163.com (Y.K.); wxh3225@163.com (X.W.); 18800429312@163.com (Z.W.); liyanhui3511@163.com (Y.L.); xufudeyouxiang2021@163.com (F.X.)

**Keywords:** black rice, anthocyanins, enzymatic acylation, lipid solubility, antioxidant activity, stability

## Abstract

In this study, the structure of the anthocyanin fractions isolated from black rice (*Oryza sativa* L.) was modified by the enzyme catalysis method using caffeic acid as an acyl donor. At the same time, the effects of the acylation on the lipophilicity, antioxidant activity, and stability of black rice anthocyanins were comprehensively evaluated. The structural analyses of acylated derivatives based on ultraviolet–visible spectroscopy, Fourier-transform infrared spectroscopy, ultra-high-performance liquid chromatography–high-resolution mass spectrometry, and thermogravimetric analysis revealed that caffeic acid was efficiently grafted onto the anthocyanins of black rice through an acylated reaction, while the acylation binding site was on glucoside. When the mass ratios of anthocyanins to caffeic acid were 1:1, the A319/AVis-max value of acylated anthocyanins reached 6.37. Meanwhile, the lipophilicity of acylated derivatives was enhanced. The antioxidant capacity (DPPH and FRAP) and stability (thermal, pH, and light stability) were significantly increased. Overall, the study results provide deeper insights into controlling anthocyanin homeostasis in food processing, broadening the application of colored grain products.

## 1. Introduction

Anthocyanins are flavonoid derivatives that are powerful antioxidants against aging and oxidative stress. So far, more than 600 anthocyanins have been detected in nature, mostly derived from cyanidin, petunidin, peonidin, pelargonidin, delphinidin, and malvidin [1,2,3]. The natural state of anthocyanins is in the form of glycosides, which are extensively utilized as colored pigments in various industries such as food, cosmetics, biology, pharmaceuticals, and others due to their diverse biological activities, bright and attractive colors, and high level of edible safety [4,5,6].

Grain is a general term for cereal plants or grain crops, covering a wide range of foods, including black rice, rice, corn, and other miscellaneous grains, which are rich in a variety of nutrients needed by the human body and form an important part of the human diet [7]. Among them, black rice is rich in phenolic compounds, including anthocyanin, phenolic acid, flavonoid, and dietary fiber, which is a great source of anthocyanins [8]. The primary anthocyanins of black rice included cyanidin and paeoniflorin, with a typical flavonoid C6-C3-C6 structure, which can be modified to prepare grain food with specific functionality to expand their applications [9,10].

Regardless of the excellent biological activity of anthocyanins, these pigments are unstable. Anthocyanins can be easily destroyed by different factors, such as pH, light, thermal treatment, enzymes, oxygen, etc., limiting their application to a great extent [11]. Therefore, improving the stability of anthocyanins has been the focus of many researchers. Accumulating studies have indicated that glycosyl in anthocyanins can combine with the acylation donor to form acylated anthocyanins, exhibiting excellent stability [12,13]. The primary sites for the acylation reaction of anthocyanin are the units of C3 and C5 in the glycosyl group, or C3, C6 [14]. Higher steric hindrance is related to the increased stability of acylated anthocyanins, which likely protects the anthocyanins from hydration. This occurs as the aglycone cannot be attacked by water, thus preventing the chalcone from forming. Furthermore, acylated anthocyanins, especially in acidic and neutral environments, exhibit excellent stability over a wide range of pH values [15].

Recently, the acylation of anthocyanins has attracted much attention. Lv et al. [16] acylated the black rice anthocyanins using octenyl succinate anhydride in pure ethanol and evaluated their stability and color change. Dini et al. [17] found that acylation reactions with aromatic carboxylic acids improved the thermal stability and light-resistivity of anthocyanins. However, the mechanism of enzymatic acylation with carboxylic acid as a donor for the structural stabilization of anthocyanins has not yet been fully elucidated. Therefore, in this study, the anthocyanins were extracted from black rice and acylated by caffeic acid in the presence of Novozyme 435 to understand the relationship between the molecular structure of acylated derivatives and their stability and antioxidant capacity. This research will provide insights to broaden the application of black rice anthocyanins in the food coloring and healthcare industries, as well as the development of new cereal foods.

## 2. Materials and Methods

### 2.1. Materials

Black rice (*Oryza sativa* L.) was obtained by Heilongjiang Beichun Agricultural Products Development Co., Ltd. (Mudanjiang, China). Lipase Novozyme 435 was supplied by the Zhejiang Novocata Biotechnology Co., Ltd. (Hangzhou, China). Caffeic acid (assay > 98%), 2,2′-azino-bis (3-ethylbenzothiazoline-6-sulfonic acid) diammonium salt (ABTS), and 2-diphenyl-1-picrylhydrazyl (DPPH) were purchased from Shanghai Yuanye Biotechnology Co., Ltd. (Shanghai, China). Other chemicals were analytical grade.

### 2.2. Anthocyanin Extraction

The method described by Hao et al. [18] was used to prepare anthocyanin extracts from black rice with some modifications. The black rice flour was dissolved in 1% hydrochloric acid/pure ethanol/water (0.5:50:50, *v*/*v*/*v*) (1:10 *w*/*v*) and extracted twice at 30 °C for 2 h. The supernatants were collected after centrifuging at 4000 r/min for 10 min. A vacuum evaporator was used to remove residual ethanol from the supernatant. Then, the crudely extracted black rice anthocyanins and AB-8 macroporous adsorption resin were mixed at a mass ratio of 5:1 for 24 h at room temperature. It was subsequently recovered using 70% ethanol (containing 1% HCl). The anthocyanin extracts were obtained by evaporation of the ethanol eluent under a vacuum and, finally, freeze-drying to produce anthocyanin powders.

### 2.3. Anthocyanin Acylation

The acylation of anthocyanins was performed by the method described by Zheng et al. [19] with slight modifications. A mixture (300 mg) of anthocyanins (AN) and caffeic acid (CA) with mass ratios of 1:0, 2:1, 1:1, and 1:2, respectively, was placed in a 250 mL conical flask, and 5 mL of distilled water was added to mix completely. The black rice anthocyanins and their derivatives were labeled AN/CA 1:0, AN/CA 2:1, AN/CA 1:1, and AN/CA 1:2, respectively. A total of 15 g of dry-activated 4A zeolite was mainly used in the absorption of water in the reaction system. Later, the mixture was mixed with tert-butyl alcohol (60 mL) and lipase Novozyme 435 (30 mg) and stirred at 25 °C for 24 h. Filtration was used to stop the reaction and remove the zeolite and enzyme. Organic reagents were removed by evaporation under vacuum and were freeze-dried for use.

### 2.4. Ultraviolet–Visible (UV–Vis) Analysis

The UV–vis analysis of black rice anthocyanins and their derivatives was performed by a Puruisi UV-18 ultraviolet and visible spectrophotometer (Tianjin Puruisi Instrument Co., Ltd., Tianjin, China). Briefly, the sample of anthocyanins was dissolved in methanol to obtain a 1.0 mg/mL solution, and stirred to sufficiently dissolve. Then, the UV–vis absorption measurements were recorded at a 1 nm sampling interval.

### 2.5. FTIR Analysis

Spectrum 100 FTIR spectrometer (PerkinElmer Co., Ltd., Shelton, CT, USA) was used to measure the chemical structure of black rice anthocyanins and their derivatives. The black rice anthocyanins and their derivatives were blended with potassium bromide (KBr) at a 1:100 (*w*/*w*) ratio and compressed into pellets. The wavenumber range was 4000 cm^−1^ to 800 cm^−1^, with a resolution of 4 cm^−1^ and 32 scans per sample.

### 2.6. Thermogravimetric Analysis (TGA)

The thermal weight loss value of black rice anthocyanins and their derivatives during heating were studied using a Q50 TGA analyzer (TA Instruments Co., Ltd., New Castle, DE, USA). Heating was carried out to 400 °C under a nitrogen atmosphere at a ramp rate of 20 °C/min. The first derivatives of the weight loss curve thermograms were calculated (DTG curves).

### 2.7. Ultra-High-Performance Liquid Chromatography–High-Resolution Mass Spectrometry/Mass Spectrometry (UHPLC–HR-MS/MS) Analysis

Agilent UHPLC-HR-MS/MS system (Thermo Fisher Scientific Inc., Waltham, MA, USA) was used to analyze the structure of the acylated anthocyanins and their derivatives. A Hypersil GOLD C18 column (length 100 nm × internal diameter 2.1 mm) with a particle size of 1.9 μm was installed in the UHPLC system. The following gradient conditions were used to elute acylated anthocyanin with 0.1% formic acid in water (A) and acetonitrile (B): 0–2 min, 5% B; 2–19 min, 5–90% B; 19–23 min, 90–95% B; 23–25 min, 5% B. The column temperature and flow rate were 40 °C and 0.3 mL/min, respectively. The following operating conditions were used for mass analysis in positive ion mode: scan range, 0–1000 *m*/*z*; capillary voltage, 3.8 kV; capillary temperature, 320 °C; dry gas flowing, 10.0 L/min and 10 μL injection volume.

### 2.8. Evaluation of the Lipophilicity, Antioxidant Activity and Stability

#### 2.8.1. Determination of the Lipophilicity

According to Xie et al. [20], the octanol-water partition coefficient (*logP*) was used to evaluate the lipophilicity of the anthocyanins and acylated derivatives. Briefly, the samples were dissolved in acidified water (2% HCl) saturated with n-ocanol, and the absorbance at 520 nm was measured (*A*_0_). Then, double the amount of octanol-saturated acidified water was added, and the mixture was centrifuged for 10 min at 3000 rpm after being vigorously shaken for 1 h. At 520 nm, the absorbance (*A_X_*) of the upper layer of octanol was determined. The following equation was used to calculate the octanol/water partition coefficient:

$$\log P=\log A_{X}/\left(A_{0}-A_{X}\right)$$

#### 2.8.2. Antioxidant Assays

The 2,2-Diphenyl-1-picrylhydrazyl (DPPH) and 2,2-azino-bis-3-ethylbenzothiazoline-6-sulfonic acid (ABTS) radical scavenging assay and ferric reducing antioxidant power (FRAP) assay were used to determine the antioxidant capacity. The method described by Sahreen et al. [21] was used to determine the DPPH and ABTS scavenging assays. The absorbance of DPPH and ABTS radical scavenging activity was measured at 517 nm and 734 nm, respectively, using a spectrophotometer. The following equation was used to calculate the scavenging rate:

$$\mathrm{Scavenging}\;\mathrm{rate}\;\left(\%\right)=\left(1-A/A_{0}\right)\times 100$$

where *A* represents the sample absorbance, and *A*_0_ represents the absorbance of the standard solution without an added sample.

The FRAP assay was performed according to the method reported by Thaipong et al. [22] with some modifications. The FRAP stock solution was made with a volume ratio of 10:1:1 of sodium acetate buffer (0.3 mol/L, pH 3.6), TPTZ solution (0.01 mol/L with 0.04 mol/L HCl), and FeCl_3_ solution (0.02 mol/L), respectively. Later, 3.98 mL of FRAP stock solution was mixed with 0.02 mL of the sample and stirred at room temperature for 30 min. Sample absorbance was measured at 593 nm. Within the range of 0 and 25 mM Trolox, the standard curve was linear. The results were represented as mM Trolox equivalents (TE)/mg anthocyanin mass.

#### 2.8.3. Stability Assays

The effect of temperature on the thermal stability of black rice anthocyanins and their derivatives was evaluated according to the method reported by Swer and Chauhan [11] with some modifications. Briefly, 1 mg/mL of anthocyanins was made. The solutions were added to test tubes with stoppers and heated at 50 °C, 60 °C, 70 °C, 80 °C and 90 °C, respectively. After cooling to room temperature, the absorbance was immediately measured at 520 nm in a spectrophotometer. Meanwhile, the same solvent was used as blank. The absorbance of anthocyanins was determined each hour for 5 h continuously. The first-order reaction rate constant (k) and half-lifetime (*t*_1/2_) were calculated by the following equations:

(1) $$\frac{C}{C_{0}}=e^{-\mathrm{kt}}$$

(2) $$t_{1/2}=\ln2/k$$

where *C* and *C*_0_ are the anthocyanin content of the sample at a particular time and the initial content, respectively, k is the rate constant (per unit time), and t is the time (in hours).

The dependence of the degradation rate constants on temperature was described by the Arrhenius model:

(3) $$\mathrm{lnk}={\mathrm{lnk}}_{0}-E_{0}/\mathrm{RT}$$

where k_0_ is the initial value of the rate constant for degradation (1/min), R is the universal gas constant, T is the absolute temperature (K), and *E*_0_ is the activation energy (kJ/mol). The slope of the ln(k) vs. 1/T plot can be employed for calculating the *E*_0_.

The pH stability (pH 3.0, 4.0, 5.0, 6.0, and 7.0) of black rice anthocyanins and their derivatives was investigated. Anthocyanin (1 mg/mL) was made at 25 °C and added to test tubes. The absorbance was measured using a spectrometer at 520 nm. The absorbance of anthocyanin was determined each hour for 5 h continuously.

The light stability of black rice anthocyanins and their derivatives was assayed. A total of 1 mg/mL of anthocyanin was made and divided into two parts. One part is sheltered from light, and one part is exposed to ultraviolet light (UV intensity ≥ 90 uw/cm^2^). After 0, 1, 2, 3, 4, and 5 h, a spectrophotometer was used to determine the change in color intensity by measuring the absorbance at 520 nm, respectively.

### 2.9. Statistical Analysis

Each experiment was repeated three times. The mean ± standard deviation (SD) was used to express the results. SPSS 22.0 (SPSS Inc., Chicago, IL, USA) was used for one-way analysis of variance (ANOVA). The graphs were created in Origin 8.5 (Origin Institute Inc., Victoria, TX, USA). *p* < 0.05 was considered statistically significant.

## 3. Results and Discussion

### 3.1. UV–Vis Analysis

**Figure 1:**
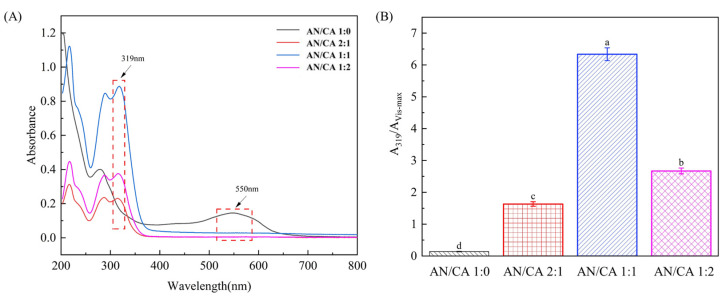
UV–vis spectra (**A**) and degree of acylation (**B**) of black rice anthocyanins and their derivatives. AN/CA 1:0, AN/CA 2:1, AN/CA 1:1, and AN/CA 1:2 represent mass ratios of anthocyanins to caffeic acid of 1:0, 2:1, 1:1, and 1:2, respectively. This explanation also applies to the following figures. Values with different letters in the same graph differ significantly (*p* < 0.05).

The effects of caffeic acid grafting on the molecular structure of black rice anthocyanins were investigated using UV–vis spectroscopy, and the results are shown in Figure 1. Two absorption bands at 280 nm and in the range of 500–550 nm were ascribed to the characteristic structure of anthocyanins. The acylation caused slight shifts, which caused the absorption maximum of anthocyanins to move from 280 nm to 289 nm. In addition, the absorption band in the range of 300–350 nm was attributed to the acylated structure of anthocyanin. The increased peak intensity of the acylated derivatives at 319 nm indicates that the acylation degree increased. Notably, the absorption peak (A319) ratio at this wavelength to the maximum wave intensity in the visible light region (A_Vis-max_) could characterize the degree of acylation of the anthocyanins [23]. The A_319_/A_Vis-max_ value of black rice anthocyanin (0.1397) increased to 1.63, 6.37, and 2.67, respectively, after grafting with caffeic acid. These results confirmed that caffeic acid was successfully grafted onto the molecules of black rice anthocyanin through the acylation reaction.

### 3.2. FTIR Analysis

**Figure 2:**
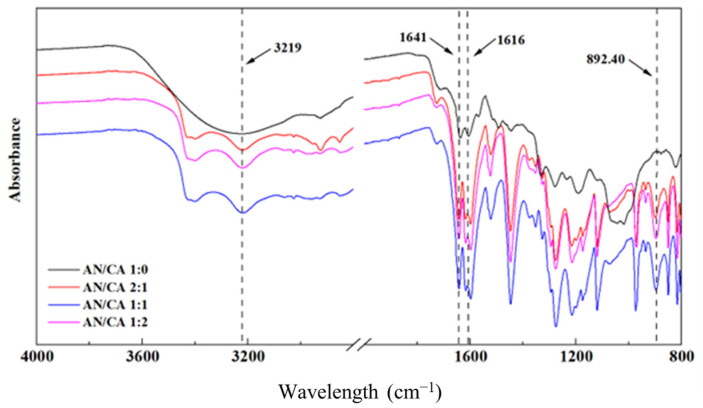
FTIR spectra of black rice anthocyanins and their derivatives.

The FTIR spectra of black rice anthocyanins and their derivatives are illustrated in Figure 2. The glycosides in the anthocyanins and numerous -OH stretching vibrations on the aromatic ring were the causes of the high absorption of black rice anthocyanins at 3226.81 cm^−1^. The caffeic acid was grafted on the black rice anthocyanins, and the hydroxyl groups in the structure of acylated derivatives increased, resulting in a wider and deeper waveform [24]. The absorption peak of the acylated derivatives shifted from 3226.81 cm^−1^ to 3219 cm^−1^, the wave seal became wider, and the wave peak increased compared with black rice anthocyanins. The peak located at 2929.82 cm^−1^ was attributed to -CH_3_, -CH_2_, and -CH [13]. The C=C vibration of the benzene skeleton in anthocyanins and caffeic acid was responsible for the characteristic peak at 1522 cm^−1^. The absorption band at 1300–1000 cm^−1^ was due to the stretching vibration of C-O, and slight blue shifts were observed at the absorption peaks of 1278.10 cm^−1^, 1188.90 cm^−1^, and 1119.00 cm^−1^ after acylation with caffeic acid, which might be attributed to the C-O-C stretching vibration in the sugar ring. The above analysis results suggested that anthocyanins could maintain their structural stability during the acylation reaction. The intensity of the characteristic peaks at 1641 cm^−1^ and 1616 cm^−1^ gradually increased with acylation. Evidently, the caffeic acids grafted onto black rice anthocyanins through the acylation reaction introduced abundant C=O groups in anthocyanins, therefore increasing the peak intensity [25]. Meanwhile, the intensity of the absorption peak at 892.40 cm^−1^ continued to increase, which could be the bending vibration of H-C=C in caffeic acid. This might have happened because the caffeic acid reacted with -OH of glycosyl in anthocyanin, generating C=C-COOAr (Ar refers to the aromatic nucleus). After the acylation reaction, the many hydroxyl groups and phenyl groups were introduced onto black rice anthocyanins, and the acyl aromatic ring underwent sandwich-type stacking on the planar pyrylium ring, which enhanced the stability of anthocyanins [26]. These results confirmed that caffeic acid was authentically grafted onto the black rice anthocyanins.

### 3.3. TGA Thermal Stability

**Figure 3:**
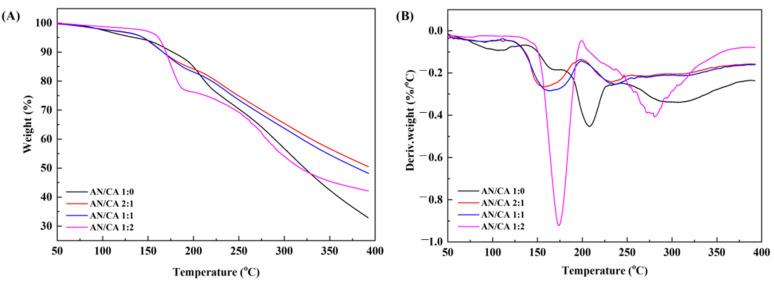
TGA (**A**) and DTG (**B**) curves of black rice anthocyanin and their derivatives.

Thermodynamic analysis can provide a deeper insight into anthocyanin thermal stability. Figure 3 demonstrates the TGA (A) and DTG (B) curves of black rice anthocyanins and their derivatives. The anthocyanin degradation could be divided into three phases according to the TGA and DTG curves. The first decomposition stage for black rice anthocyanins and their derivatives appeared at 100 °C and 125–200 °C, respectively, due to the presence of a small amount of absorbed and bound water [27]. The loss of black rice anthocyanins and their derivatives samples in the second stage was significant (*p* ≤ 0.05). The maximum weight loss occurred at approximately 205 °C for anthocyanins and 230 °C or 280 °C for the acylated derivatives, showing compound decomposition of anthocyanins [28]. In the third stage, the weight loss for anthocyanins occurred at approximately 300 °C, which was insignificant for the acylated derivatives. The acylated derivatives showed stronger thermal stability than black rice anthocyanins at higher temperatures, indicating that the acylation reaction had a protective effect on the structure of anthocyanins. Notably, compared to black rice anthocyanins, the degradation of the acylated derivatives for AN/CA 2:1, AN/CA 1:1, and AN/CA 1:2 migrated into higher regions, but the thermal weight loss value in the first stage reached the maximum for AN/CA 1:2. It was reported that the thermal decomposition temperature of caffeic acid was between 153 and 241.2 °C. This may be due to the thermal degradation of excess caffeic acid in the AN/CA1:2.

### 3.4. UHPLC–HR-MS/MS Analysis

**Figure 4:**
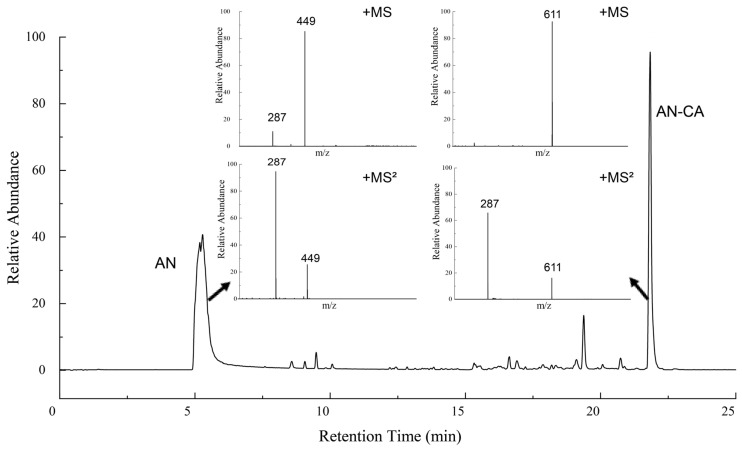
The UHPLC-MS spectra of black rice anthocyanins and their derivatives.

Figure 4 shows the UHPLC-MS spectra of anthocyanins before and after the acylation reaction. The structure of the acylation reaction products was studied in the positive ion mode. This is combined with previous research in the laboratory, which found that anthocyanins were detected at a retention time of 5.27 min, where the daughter ion at *m*/*z* 287 in the MS^2+^ spectra originated from the parent ion at *m*/*z* 449, which was a typical ion peak for anthocyanins. The base peak at *m*/*z* 449 represented the cyanidin-3-glucoside [29]. In MS^+^ mass spectrometry, a new molecular ion signal peak of *m*/*z* 611 at retention time 21.82 min, which happened to be the total molecular weights of caffeic acid and anthocyanins, combining with the UV and FTIR data indicated that the reaction with caffeic acid resulted in the formation of an acylation product. Notably, according to the MS^2+^ of AN-CA, the *m*/*z* of the other two ion signal peaks were 287 and 611, respectively. The observation of fragment ions in the same position as the AN parent ion suggested that the acylation was regioselective for the glycosidic portion and occurred preferentially on the hydroxyl group in the glycoside [23].

### 3.5. Determination of the Lipophilicity

**Figure 5:**
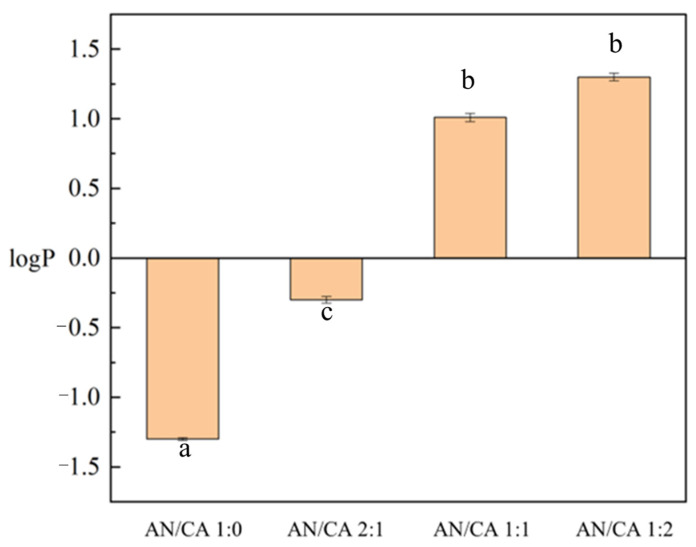
Determination results of the lipophilicity for black rice anthocyanins and their derivatives. Values with different letters in the same graph differ significantly (*p* < 0.05).

The results of the octanol/water partition coefficient (*logP*) of black rice anthocyanins and their derivatives are shown in Figure 5. The *logP* of black rice anthocyanins significantly increased from negative to positive after acylation with caffeic acid, indicating a characteristic shift from hydrophilicity to lipophilicity. After acylation, the *logP* of anthocyanins increased, presumably due to the acylation reaction resulting in acylation products, which enhanced the lipophilicity of anthocyanins. Paulina et al. [30] found that acylation of anthocyanins reduced the fluidity of the membrane hydrophobic and significantly increased the affinity of the membrane, so the above phenomenon also broadened the ideas for the study of improving the bioavailability of black rice anthocyanins.

### 3.6. Antioxidant Capacity

**Figure 6:**
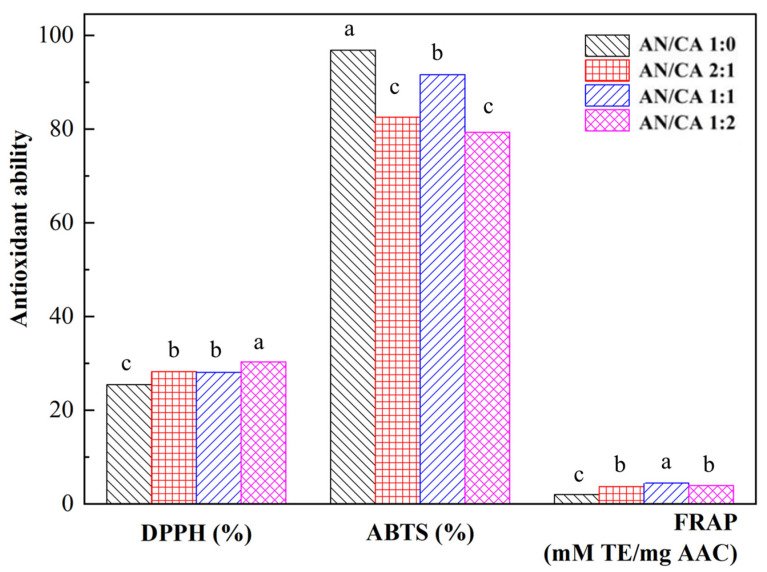
The DPPH, ABTS, and FRAP for black rice anthocyanins and their derivatives. Values with different letters in the same graph differ significantly (*p* < 0.05).

The antioxidant capacity of black rice anthocyanins and their derivatives is shown in Figure 6. The acylated derivatives (AN/CA 2:1, AN/CA 1:1, and AN/CA 1:2) exhibited good antioxidation activity in DPPH assays, the DPPH clearances of 28.20%, 28.10%, and 30.35%, respectively. Nevertheless, the acylated derivatives were slightly inferior to black rice anthocyanins in the ABTS radical scavenging assay. This indicated that the DPPH radical had good lipid solubility and stability in the organic solvent ethanol, the poorer water solubility of the ABTS radical, and the acylation reaction enhanced the liposolubility of anthocyanin, therefore increasing its antioxidant capacity in organic solvents [31]. Additionally, the presence of many hydroxyl groups in caffeic acid gave it good antioxidant activity, and the DPPH clearance was slightly better after grafting the caffeic acid onto anthocyanins [13]. The FRAP approach yielded a total antioxidant capacity for the acylated derivatives that was twice that of black rice anthocyanins, indicating that acylation enhanced the antioxidant capacity of black rice anthocyanins.

### 3.7. Stability Assays

#### 3.7.1. Thermal Stability

**Figure 7:**
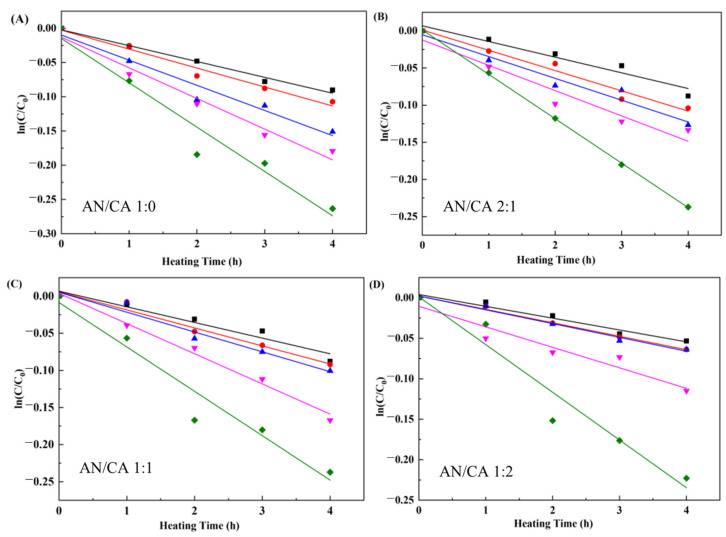
Degradation of black rice anthocyanin and their derivatives treated at different temperatures (■ 50 °C, ● 60 °C, ▲ 70 °C, ▼ 80 °C, ◆ 90 °C).

The influence of temperature from 50 °C to 90 °C on the stability of black rice anthocyanins and their derivatives, respectively, was investigated. Figure 7 displayed the change in ln(*C*/*C*_0_) with respect to heating time. The results were consistent with the previous studies reporting that the degradation of black rice anthocyanins and their derivatives followed a first-order reaction model.

Table 1 summarizes the kinetic parameters. As expected, the *k* value increased, and *t*_1/2_ decreased gradually with increased heating temperature, implying that heat treatment promoted the degradation of anthocyanins. The anthocyanin stability could be enhanced after being acylated through self-association reactions and intramolecular and intermolecular co-pigmentation [32]. This study showed that the acyl donors were arranged on one side of the pyran ring after the acylation reaction occurred, preventing nucleophilic attack by water and allowing only weak intermolecular interactions, protecting the molecular structure of the acylated anthocyanins [23,33]. Notably, the *k* values decreased gradually, and the *E*_0_ values improved as the caffeic acid content increased. This suggested that higher caffeic acid content led to a greater energy barrier required during degradation. The improvement of anthocyanin stability in samples (AN/CA 2:1 and AN/CA 1:1) by acylation was mainly attributed to the reduction of acyl group polarity and site resistance, which protected the chromophore from nucleophilic attack by water [34]. Combined with the previous acylation degree determination results, it was speculated that in addition to the above reasons for the increased stability of sample AN/CA 1:2, co-pigmentation of acylated anthocyanins with excess caffeic acid in the reaction system effect also prevented the thermal degradation of the acylated anthocyanins [35].

#### 3.7.2. pH Stability

**Figure 8:**
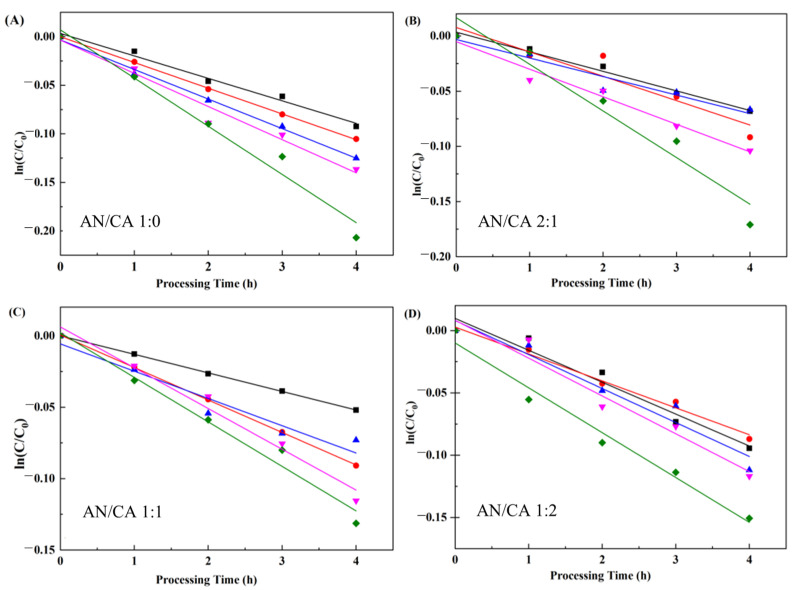
Degradation of black rice anthocyanins and their derivatives treated under conditions of different pH (■ pH 3, ● pH 4, ▲ pH 5, ▼ pH 6, ◆ pH 7).

The stability of black rice anthocyanins and their derivatives was assessed at different pH ranging from 3.0 to 7.0. Figure 8 describes the degradation of black rice anthocyanins and their derivatives under various pH with kinetic parameters shown in Table 2. As shown in Figure 8 and Table 2, the black rice anthocyanins and their derivatives had higher *k* values and lower *t*_1/2_ values at high-pH treatments, implying lower stability of the sample at higher pH values. This was attributed to the fact that the electron-deficient form of anthocyanins was affected by the charge when the environmental pH changed, and the chemical structure changed accordingly. These results were consistent with the previous study results [16]. All acylated anthocyanins had greater pH stability than black rice anthocyanins. This indicated that the pyran ring of anthocyanins reduced the susceptibility to hydrophilic group attack because of acyl stacking, preventing the formation of achromatic pseudobases and chalcone structures from black rice anthocyanin [36]. Meanwhile, k of the acylated derivatives slightly increased with increased content of caffeic acid and remained lower than that of black rice anthocyanins. This result confirmed that acylation improved the pH stability of anthocyanin.

#### 3.7.3. Light Stability

**Figure 9:**
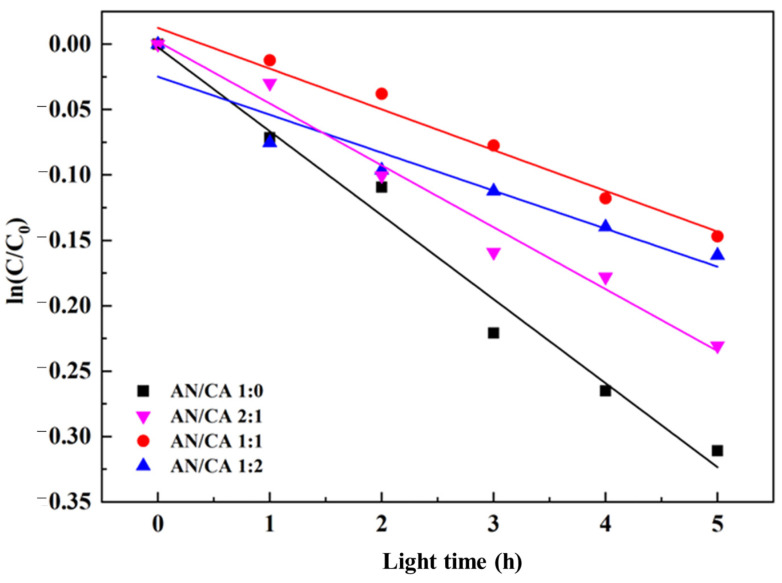
Degradation of black rice anthocyanins and their derivatives under UV light.

As shown in Figure 9 and Table 3, the rate of degradation of the anthocyanins in black rice was higher than that of the acylated derivatives when exposed to UV radiation. Acylated derivatives may be attributed to the changes in the structural conformation and the presence of intramolecular and/or intermolecular stacking (such as acylation, self-association, and co-pigmentation) of anthocyanins, as well as the stable acryl ring in caffeic acid structure, which protected anthocyanins from degradation factors [19,37]. Additionally, the increasing caffeic acid content also increased the light stability of the acylated derivatives. This may be the result of the additional caffeic acid providing intermolecular co-pigmentation, therefore increasing the stability of black rice anthocyanins.

## 4. Conclusions

The stability of black rice anthocyanins has an important implication for their application as a natural pigment or functional ingredient in food. Therefore, in this study, the black rice anthocyanins were acylated with caffeic acid to address their poor stability. The analysis of UV, FTIR, and UHPLC-HR-MS/MS showed that the acylation reaction took place at the hydroxyl site of glucoside, forming an acylated derivative of anthocyanins. Acylation with caffeic acid enhanced the lipophilicity of black rice anthocyanins. The antioxidant activity of the acylated derivatives in the hydrophilic systems (ABTS assays) decreased, but the clearance ratio in DPPH and the total antioxidant capacity assay increased. Compared with black rice anthocyanins, the degradation kinetic parameters of the acylated derivatives showed a trend of lower degradation rate k and longer degradation half-life *t*_1/2_. The stability (temperature, pH, and light) of the acylated derivatives increased. This study establishes an acylation route for the synthesis of highly stable anthocyanin-based pigments, further broadening the application of black rice anthocyanins in functional foods or as novel functional colorants.

## Figures and Tables

**Table 1:** Thermal degradation kinetics data of black rice anthocyanin and their derivatives. Values with different letters in the same graph differ significantly (*p* < 0.05).

*T* (°C)	*k* (10^−2^/h)				*t*_1/2_ (h)				*E*_0_ (kJ/mol)			
	AN/CA 1:0	AN/CA 2:1	AN/CA 1:1	AN/CA 1:2	AN/CA 1:0	AN/CA 2:1	AN/CA 1:1	AN/CA 1:2	AN/CA 1:0	AN/CA 2:1	AN/CA 1:1	AN/CA 1:2
50	2.47 ± 0.18 ^a^	1.61 ± 0.43 ^b^	1.36 ± 0.61 ^b^	1.12 ± 0.41 ^b^	28.04 ± 2.12 ^d^	43.02 ± 0.25 ^c^	50.93 ± 0.72 ^b^	61.95 ± 8.26 ^a^	23.69 ± 1.22 ^c^	25.52 ± 1.31 ^c^	34.47 ± 1.53 ^b^	39.27 ± 1.82 ^a^
60	2.91 ± 0.41 ^a^	2.65 ± 0.35 ^a^	1.92 ± 0.76 ^b^	1.46 ± 0.28 ^b^	23.80 ± 3.16 ^c^	26.18 ± 3.65 ^c^	36.16 ± 1.17 ^b^	47.56 ± 0.75 ^a^				
70	4.39 ± 0.73 ^a^	3.37 ± 0.57 ^b^	2.18 ± 0.89 ^c^	1.50 ± 0.32 ^c^	15.80 ± 2.64 ^d^	20.58 ± 3.77 ^c^	31.74 ± 1.94 ^b^	46.14 ± 2.26 ^a^				
80	5.47 ± 0.92 ^a^	4.29 ± 0.74 ^b^	3.83 ± 0.29 ^b^	3.43 ± 0.46 ^b^	12.67 ± 2.10 ^c^	16.16 ± 3.10 ^b^	18.11 ± 1.40 ^a^	20.23 ± 3.91 ^a^				
90	7.51 ± 0.76 ^a^	5.87 ± 0.15 ^b^	5.69 ± 0.98 ^b^	5.58 ± 0.18 ^b^	9.23 ± 1.44 ^b^	11.81 ± 0.31 ^a b^	12.17 ± 2.25 ^a^	12.43 ± 1.75 ^a^				

**Table 2:** Degradation kinetics data of black rice anthocyanins and their derivatives under conditions of different pH. Values with different letters in the same graph differ significantly (*p* < 0.05).

pH Value	*k* (10^−2^/h)				*t*_1/2_ (h)			
	AN/CA 1:0	AN/CA 2:1	AN/CA 1:1	AN/CA 1:2	AN/CA 1:0	AN/CA 2:1	AN/CA 1:1	AN/CA 1:2
3	2.04 ± 0.37 ^a^	1.50 ± 0.28 ^b^	1.88 ± 0.37 ^a^	1.93 ± 0.30 ^a^	34.00 ± 2.48 ^c^	46.10 ± 0.8 ^a^	36.67 ± 0.77 ^b^	35.84 ± 2.42 ^c^
4	2.65 ± 0.45 ^a^	1.88 ± 0.40 ^b^	2.10 ± 0.69 ^a b^	2.24 ± 0.03 ^a b^	26.15 ± 0.45 ^c^	36.97 ± 0.99 ^a^	32.96 ± 0.32 ^b^	31.00 ± 0.32 ^b^
5	3.33 ± 0.34 ^a^	1.91 ± 0.25 ^c^	2.30 ± 0.37 ^b^	2.42 ± 0.07 ^b^	20.84 ± 1.98 ^d^	36.23 ± 1.23 ^a^	30.18 ± 2.56 ^b^	28.66 ± 0.66 ^c^
6	3.64 ± 0.54 ^a^	2.20 ± 0.11 ^b^	2.41 ± 0.37 ^b^	2.56 ± 0.67 ^b^	19.08 ± 2.54 ^c^	31.59 ± 1.20 ^a^	28.77 ± 3.01 ^b^	27.09 ± 2.26 ^b^
7	4.48 ± 0.50 ^a^	3.32 ± 0.84 ^b^	3.81 ± 0.52 ^b^	4.40 ± 0.83 ^a^	15.49 ± 1.61 ^c^	23.39 ± 3.84 ^a^	18.20 ± 2.53 ^b^	15.77 ± 1.68 ^c^

**Table 3:** Degradation kinetics data of black rice anthocyanins and their derivatives at conditions of UV light. Values with different letters in the same graph differ significantly (*p* < 0.05).

Samples	*k* (10^−2^/h)	*t*_1/2_ (h)
AN/CA 1:0	6.42 ± 0.44 ^a^	10.43 ± 0.06 ^c^
AN/CA 2:1	4.73 ± 0.33 ^b^	14.65 ± 0.07 ^b^
AN/CA 1:1	3.11 ± 0.24 ^c^	22.28 ± 0.07 ^a^
AN/CA 1:2	2.90 ± 0.43 ^c^	23.90 ± 0.15 ^a^

## Data Availability

Data is contained within the article.
